# The Lacunocanalicular Network is Denser in C57BL/6 Compared to BALB/c Mice

**DOI:** 10.1007/s00223-024-01289-y

**Published:** 2024-10-16

**Authors:** Maximilian Rummler, Alexander van Tol, Victoria Schemenz, Markus A. Hartmann, Stéphane Blouin, Bettina M. Willie, Richard Weinkamer

**Affiliations:** 1 Department of Biomaterials, Max Planck Institute of Colloids and Interfaces, Am Mühlenberg 1, 14476 Potsdam, Germany; 2 1st Medical Department Hanusch Hospital, Ludwig Boltzmann Institute of Osteology at Hanusch Hospital of OEGK and AUVA Trauma Centre Meidling, Vienna, Austria; 3 Research Center, Shriners Hospital for Children, Montreal, Canada; 4 Faculty of Dental Medicine and Oral Health Sciences, McGill University, Montreal, Canada; 5 Department of Operative and Preventive Dentistry, Charité-Universitätsmedizin - Berlin, Berlin, Germany

**Keywords:** Networks, Bone, Osteocytes, Mechanobiology, Image analysis

## Abstract

The lacunocanalicular network (LCN) is an intricate arrangement of cavities (lacunae) and channels (canaliculi), which permeates the mineralized bone matrix. In its porosity, the LCN accommodates the cell network of osteocytes. These two nested networks are attributed a variety of essential functions including transport, signaling, and mechanosensitivity due to load-induced fluid flow through the LCN. For a more quantitative assessment of the networks’ function, the three-dimensional architecture has to be known. For this reason, we aimed (i) to quantitatively characterize spatial heterogeneities of the LCN in whole mouse tibial cross-sections of BALB/c mice and (ii) to analyze differences in LCN architecture by comparison with another commonly used inbred mouse strain, the C57BL/6 mouse. Both tibiae of five BALB/c mice (female, 26-week-old) were stained using rhodamine 6G and whole tibiae cross-sections were imaged using confocal laser scanning microscopy. Using image analysis, the LCN was quantified in terms of density and connectivity and lacunar parameters, such as lacunar degree, volume, and shape. In the same tibial cross-sections, the calcium content was measured using quantitative backscattered electron imaging (qBEI). A structural analysis of the LCN properties showed that spatially denser parts of the LCN are mainly due to a higher density of branching points in the network. While a high intra-individual variability of network density was detected within the cortex, the inter-individual variability between different mice was low. In comparison to C57BL/6J mice, BALB/c mice showed a distinct lower canalicular density. This reduced network was already detectable on a local network level with fewer canaliculi emanating from lacunae. Spatial correlation with qBEI images demonstrated that bone modeling resulted in disruptions in the network architecture. The spatial heterogeneity and differences in density of the LCN likely affects the fluid flow within the network and therefore bone’s mechanoresponse to loading.

**Supplementary Information:**

The online version contains supplementary material available at 10.1007/s00223-024-01289-y.

## Introduction

The lacunocanalicular network (LCN) is an intricate (sub-)microscopic porosity that permeates bone. The lacunae of the network accommodate the cell bodies of osteocytes, while in the canaliculi the osteocytes’ cell processes reside. The osteocytes themselves organize in the form of a network. This cell collective contributes essentially to bone health including its function as an endocrine gland [1]. Despite substantial progress in live cell imaging [2], their encasement in mineralized tissue makes an in vivo observation of osteocytes particularly challenging. Instead of investigating osteocytes directly, a promising research target is to study their housing cavity, i.e., the lacunocanalicular network. All the more, as the fluid filled space within the LCN but outside of the osteocyte network seems to be crucial for bone’s mechanosensitivity. Evidence is accumulating in support of the Fluid Flow Hypothesis [3] which states that load-induced fluid flow through the LCN creates shear and drag forces [4–7], which the osteocytes are able to sense. This information is then processed by osteocytes to orchestrate bone remodeling [8]. Also, the role of osteocytes in mineral homeostasis via perilacunar/pericanalicular remodeling is related to the architecture of the LCN [9, 10]. Although the volume fraction of canaliculi is only about 0.5% [11–13] due to a diameter of canaliculi of approximately only 300 nm [11], one cubic centimeter of human osteonal bone contains 74 km of canaliculi [14] generating a surface area of about 700 cm^2^. As a consequence of the high network density, 80% of the bone is closer than 2.77 µm to the next canaliculus or lacuna [14]. In sum, the LCN provides excellent access for the osteocytes to the surrounding mineralized matrix [15].

To obtain the architecture of the LCN three-dimensional imaging techniques [16] have to be applied including the use of electrons, X-rays or light for imaging. Widely used techniques include focussed ion beam-scanning electron microscopy (FIB-SEM) [12, 17], synchrotron based nanotomography [11, 13, 18], and confocal laser scanning microscopy [2, 14]. The high spatial resolution of the first two techniques allows a measurement of the diameter of single canaliculi, but has the downside of a restricted imaged volume including only a few lacunae with emanating canaliculi [12]. In contrast, extensive confocal imaging and stitching multiple image stacks allows to cover the complete cross-section of a mouse bone [19]. The result is a description of the LCN as a spatial network [20] consisting of nodes (= branching points, i.e., lacunae and intersections between canaliculi) and edges (= branches, i.e., the canaliculi) [21, 22]. These network data can then be quantified (globally and locally), for example in terms of network density and how strong the network branches at branching points.

Such a quantitative description of the LCN is available for human osteonal bone and cortical bone from C57BL/6J mice demonstrating the much higher density of the LCN in mice [19]. Mice have become a preferred model system of bone research due to genetic similarities to humans and the wide possibilities of genetic manipulation [23]. However, different mouse strains do not only have different bone density and structure [24], but also different material properties [25] and different bone responses to ovariectomy [26] and development of osteoarthritis [27]. In current preclinical research two of the most commonly used inbred mouse strains are C57BL/6 and BALB/c. The selection between these two strains is mainly based on their immunological differences. BALB/c mice are widely used in studies on oncology [28] and inflammation [29], while the C57BL/6 mouse is often chosen to examine age-related bone loss [30]. However, it is not known which mouse model has the most appropriate LCN architecture to study one or more of the functions attributed to it. An important difference between mouse strains lies in their response to mechanical loading in terms of bone formation and resorption. An in vivo tibial loading study comparing four-month-old female mice from BALB/c and C57BL/6J mice showed differences in both the cortical and trabecular bone formation response to loading, although peak strains engendered differed by approximately 20 percent [31]. We compared these two genetic strains and showed that when using the same in vivo tibial loading protocol, higher strains were required to elicit a similar cortical and trabecular bone formation response in 10-week-old female BALB/c mice compared to C57BL/6J mice [32].

Having in mind the impact of the architecture of the LCN on bone’s mechanoresponse as stated by the Fluid Flow Hypothesis [3], the main aims of our study are (i) to quantitatively characterize the architecture and the spatial heterogeneities of the LCN in whole mouse tibial cross-sections of BALB/c mice and (ii) to analyze differences in LCN architecture by comparison with previously reported network properties of C57BL/6J mice [19]. For this comparison we analyzed the LCN in imaged volumes covering the whole tibial cross-sections in both limbs from five BALB/c mice. Since to the left tibiae a controlled mechanical loading was applied, additionally we can address the question whether loading results in architectural changes of the established LCN in the pre-existing cortical bone. Based on the obtained image data of the LCN, we contrast the intra-individual to the inter-individual variability of network properties. Specifically, we ask the question whether higher network densities are achieved by introducing more branching points or by increasing the number of branches emanating from the branching point. In all the tibial cross-sections, maps of the calcium content were obtained by quantitative backscattered electron imaging. These maps display regions of different Ca content separated by sharp interfaces. A spatial correlation with LCN images indicated that interfaces in the qBEI image correspond to discontinuities in the network structure. Consequently, these interfaces can be interpreted as results of discrete modeling processes. A key finding of our study is that the LCN is denser in C57BL6/6J mice compared to BALB/c mice and this higher density already manifests itself locally by more canaliculi emanating from lacunae in C57BL6/6J mice.

## Materials and Methods

### Samples

Five, 26-week-old female BALB/c mice were group housed in the animal facility at Shriners Hospital for Children-Canada with ad libitum access to food and water. Mice were group housed three per cage at controlled temperature (20 ± 2 °C) with the mice on a 12h:12h light–dark cycle with the light period beginning at 6:00 and ending at 18:00. The left tibiae of these mice underwent in vivo cyclic compressive loading using a loading device (Testbench ElectroForce LM1, TA Instruments, New Castle, USA) while the right limb served as an internal control and was not loaded. Mice were anesthetized during loading using isoflurane (2% in 1.0l/min O2). The loading protocol consisted of 216 loading cycles applied at 4 Hz, with a rest insertion of 5 s after 4 cycles [33, 34]. Loading was applied 5 days/week for 2 weeks, delivering − 10 N peak loads, which engendered 1800 *με* at the medial surface of the tibial-midshaft. We chose to examine female skeletally mature mice to avoid the effects of growth and since grouped housed male mice often fight, which can mask the effects of in vivo tibial loading [35]. Mice were sacrificed after the 2-week loading protocol by cervical dislocation. All animal experiments were carried out according to the policies and procedures approved by the local legal research animal welfare representative (McGill University animal use protocol 2016-7821).

We compared results from five BALB/c mice to results from five 26-week-old female C57BL/6J mice. It should be noted that three of the five C57BL/6J mice were used in a previous study that focussed on the fluid flow through the LCN and reported their canalicular density and pore volume fraction [19]. As previously reported, C57BL/6J mice underwent the identical loading protocol as BALB/c, except that a load level of − 11 N was applied that engendered 1200 με at the medial surface of the tibial-midshaft [19]. The target peak mechanical strain values engendered during loading were different between the two mouse strains to achieve a similar anabolic response. The processing and analyses of the tibiae was identical to the BALB/c. These animal experiments were carried out according to the policies and procedures approved by the local legal representative (LAGeSo Berlin, G0168/13).

### Embedding and Rhodamine 6G Staining

After sacrificing of the animals and dissection of the tibiae, the bones were fixed in 70% ethanol. Prior to staining and embedding, the tibiae were subjected to a graded series of ethanol (70%, 80%, 95%, and 100%—each for 24h) in order to dehydrate the samples. Afterward samples were stained using a Rhodamine 6G (Rh6G, Sigma Aldrich GmbH, Taufkirchen, Germany)/EtOH solution (0.417 g/100 ml) for 3 × 24h, changing the solution daily [36]. Post staining, tibiae were embedded in polymethyl methacrylate (PMMA). The blocks were then cut transversally in the tibial-midshaft region to a thickness of about 2 mm, around 1.5 mm above the tibia-fibular junction using a diamond wire saw with a 50 µm-wire and polished.

### Confocal Microscopy

To obtain structural information of the stained lacunocanalicular network (LCN) within the tibial cross-sections, PMMA embedded blocks were scanned using a Leica SP8 (Leica Microsystems GmbH, Wetzlar, Germany) confocal laser scanning microscope (CLSM). The microscope is equipped with a 40 × oil immersion objective (NA 1.3). Using a zoom factor of 0.75 leads to images of 1024 × 1024 pixels (pixel size = 379 nm). An argon laser at 10% the maximum output power (6.5 mW) at an operated wavelength of 514 nm was chosen to excite Rh6G, while the emission was measured in a spectral window between 550 and 650 nm with the airy 1 pinhole (67.93 µm) [19, 36]. Image stacks with a spatial z-resolution of 340 nm were taken up to a depth of 60 µm. Since the size of a tibial cross-section (~ 1.2 mm) is larger than the field of view (0.4 mm) of the microscope, up to 16 image stacks covering the whole cross-section were recorded with an overlap of about 10%. One tibial cross-section was imaged in this way for each tibia.

### Image Postprocessing and Network Analysis

The imaged stacks are stitched together using BigStitcher in ImageJ [37]. This tool allows an accurate alignment of canaliculi to ensure their continuity between different image stacks. To obtain the network topology from the 3D image stacks, we use an updated custom-made Python software package called Tool for Image and Network Analysis (TINA) [14, 19, 38]. In short the following steps are performed: (i) binarization of the dataset employing a local adaptive thresholding algorithm based on difference of Gaussians, (ii) segmentation of lacunae and canaliculi based on their size/bulkiness, (iii) skeletonization of the image dataset, (iv) smoothing of the skeletonized image data using third order smoothing splines and finally (v) translation into a network using NetworkX 1.11 [39] consisting of nodes (i.e., branching points, includes lacunae and intersection points between canaliculi) and edges (canaliculi or branches of the network) [14, 19, 36, 38].

Once a network description of the LCN is obtained we use TINA for further quantitative analysis. We evaluate canalicular density (Can.Dn [µm/ µm^3^]), the total length of canaliculi per unit volume, average node number (Nd.Nr [# of nodes/ µm^3^]), the average number of network nodes per unit volume, and average node degree (Nd.Dg [dimensionless]), the average degree of a node (i.e., the number of branches emanating from the node, not counting lacunae), within 400 µm^3^ subvolumes (7.4 × 7.4 × 7.4 µm^3^).). The size and shape of lacunae is quantitatively described by three parameters: lacunar volume (Lc.V [µm^3^]), lacunar stretch (Lc.St [dimensionless]), and lacunar oblateness (Lc.Ob [dimensionless]) [38, 40]. Describing the lacuna as an ellipsoid, lacunar stretch informs about the ratio between the longest and the shortest axis of the ellipsoid. Lacunar oblateness provides information whether the intermediate axis is more similar to the shortest axis (i.e., rod-like lacuna) or more similar to the longest axis (i.e., plate-like lacuna) [38, 40]. The lacunar degree (Lc.Dg [dimensionless]) describes the number of primary canaliculi emanating from a lacuna. Newly formed cortical bone showed overstaining with rhodamine and was therefore excluded from the analysis (Supplemental Fig. 6).

**Fig. 1:**
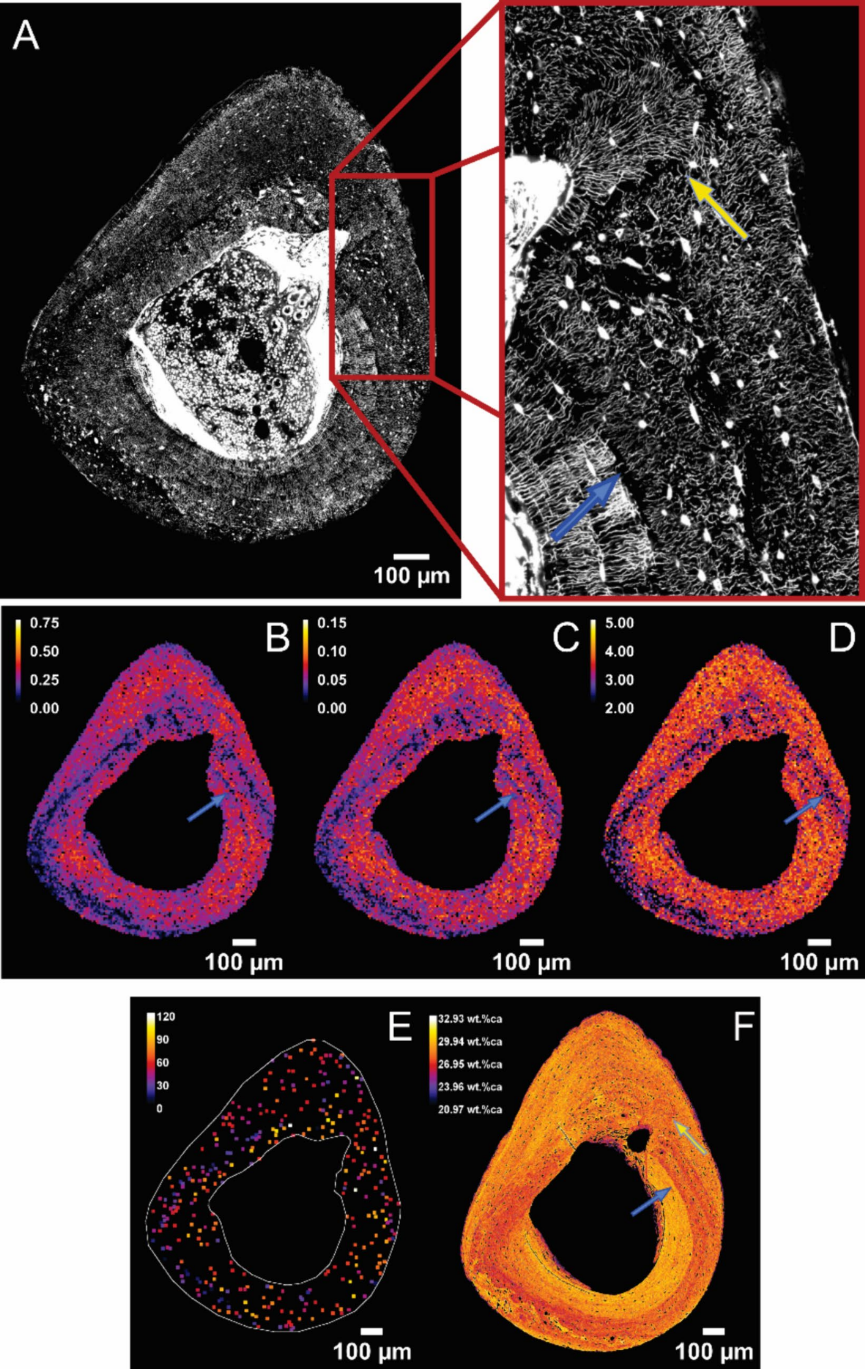
Lacunocanalicular network within a tibial cross-section of a BALB/c mouse, left, loaded tibia. **A** shows a single image of an image stack obtained by confocal microscopy where bright dots correspond to osteocyte lacunae. The zoomed inset highlights the intricate web-like structure of the LCN as well as special features such as disruptions of the network (blue arrow) and a fanning LCN architecture in the vicinity of a drifting blood vessel (yellow arrow). **B–E** show maps of network parameters obtained by evaluation of network properties within 400 µm^3^ cubic subvolumes: **B** canalicular density as length of the canaliculi per volume [µm/µm^3^], **C** the average node number, i.e., the number of branching points of the network per volume [#/µm^3^], **D** the average node degree, i.e., the average number of branches emanating form a branching point; **E** an intensity projection along the z-axis of 3.4 µm of lacunae and their degree, i.e., the number of canaliculi emanating from the lacunae. The white line shows the outline of the tibial cross-section. **F** the Ca content [wt%Ca] measured by quantitative Backscattered Electron Imaging (qBEI). The interfaces seen in the qBEI image (blue arrow) can also be spotted in the spatial maps of the LCN (**B–D**) as disruption in the network (inset of A)

An outcome of our image analysis are spatial maps of network parameters (Fig. 1B–D), where the spatial resolution is defined by the size of the subvolumes. These data are then condensed into the following plots: (i) Two different network parameters obtained from the same subvolumes were plotted against each other in an *x*–*y* plots (correlation plot). The cloud of data points was fitted by least-squares linear regression using the SciPy stats package [41] for Python. (ii) Normalized frequency histograms (i.e., probability distributions) were plotted for network and lacunar properties. Distributions including all samples for a mouse strain were smoothed using a Gaussian kernel (*σ* = 1). The average curve and the 95% confidence interval were plotted using Seaborn for Python. (iii) Independent of the division into subvolumes, the degree of all “genuine” network nodes (i.e., nodes with a degree of 3 or larger) was assessed in the whole tibial cross-section. The cumulative node degree distribution provides information for each number $x>3$, which percentage of nodes has a degree equal or larger than *x* [42]. This distribution was then fitted using an exponential decay function ${e}^{-\beta (x-3)}$ with the SciPy optimize package [41, 42].

### Segmentation Between Regions of Ordered and Unordered Network

In tibial cross-sections, regions with two conspicuously different network organizations can be observed: An “ordered” network with well-aligned canaliculi, which run close to each other. In contrast, the “unordered” network does not display patches of aligned canaliculi and in the CLSM images regions appear darker due to fewer canaliculi (Fig. 1A). To allow a most accurate separation between ordered and unordered network regions, the LCN in the image stack was first skeletonized. Then the skeletonized image stack was intensity projected along the *z*-axis (i.e., perpendicular to the cross-sectional plane) to obtain a single image (an example is shown in the Supplementary Material, Fig. S4). Finally, manual segmentation was employed to separate ordered network regions, which appear in this projected image as a denser ‘comb’-like structure, from unordered network, which have a much looser, ‘whirl’-like appearance. This manual segmentation was performed by different individuals to ensure a reasonable reproducibility of the procedure.

### Quantitative Backscattered Electron Imaging (qBEI)

To prevent charging during scanning electron microscopy, the block surfaces are coated with a thin carbon layer (approx. 20 nm) by vacuum evaporation (Agar Scientific, SEM carbon coater, Essex, UK). Quantitative Backscattered Electron Imaging (qBEI) [43, 44] was performed using a digital scanning electron microscope (Supra 40, Zeiss, Oberkochen, Germany) equipped with a four-quadrant semiconductor BE detector. The electron microscope was used with following settings: 10 mm sample-detector-distance, 130 × magnification, 20 kV acceleration voltage and a probe current between 280 and 320 pA. Each pixel has a nominal size of 0.88 × 0.88 µm^2^ and a specific gray level (256 bins, corresponding to 8 bit) which corresponds to a certain calcium content after calibration. The calibration was performed using reference samples of pure carbon and aluminum according to published standard procedures [44, 45].

### Statistics

Network parameters are reported as average over all samples ± standard deviation with *n* = 5 for each group. Comparisons between groups were assessed using a Student’s t-test, either with a paired sample t-test for comparing non-loaded and loaded BALB/c mice or an independent t-test for comparison between BALB/c and C57BL/6J mice. Linear regression was performed using a least-squares fit. Fit quality is reported using the Pearson correlation coefficient R. Intra-individual differences were assessed by the mean of standard deviations of each distribution and compared to the inter-individual differences (standard deviation of the distribution of the means). All tests have been performed in Python using the SciPy stats and optimize packages [41].

## Results

### Different Network Parameters Show Substantial Spatial Heterogeneity in the Cortex

**Fig. 2:**
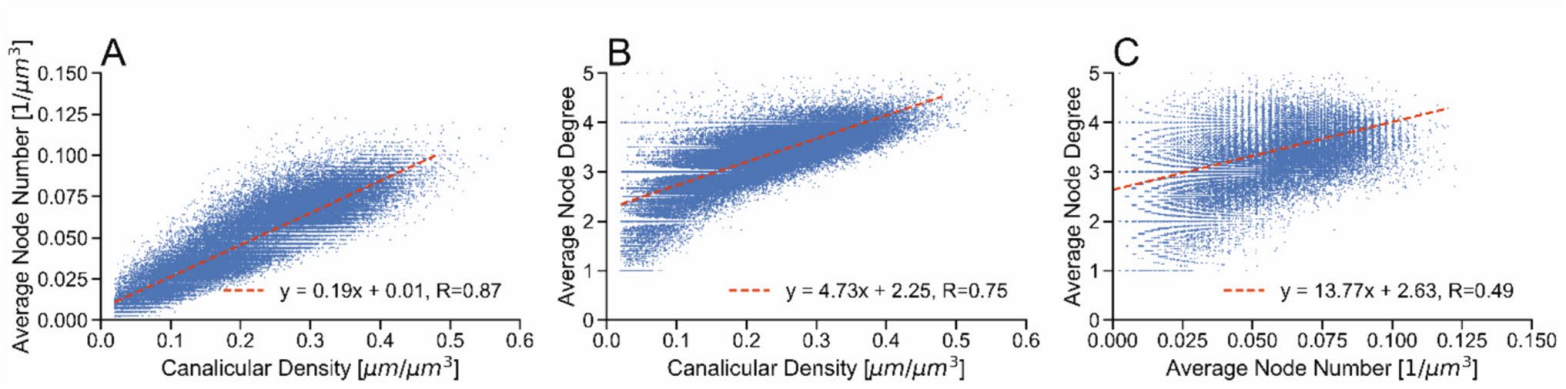
Correlation plots of network parameters for the BALB/c mouse of Fig. 1, left, loaded, tibia. **A** shows the correlation between canalicular density and average number of branching points (average node number), **B** the correlation between canalicular density and average number of branches emanating from a branching point (average node degree) and **C** the correlation of average node number and average node degree. The red line denotes a linear least squares regression; given is slope and intercept of the straight line, together with the Pearson correlation coefficient R. Patterns in the arrangement of data points as seen in Fig. 2C are due to subvolumes with only a few nodes and the mode of plotting, which spreads identical data points

**Fig. 3:**
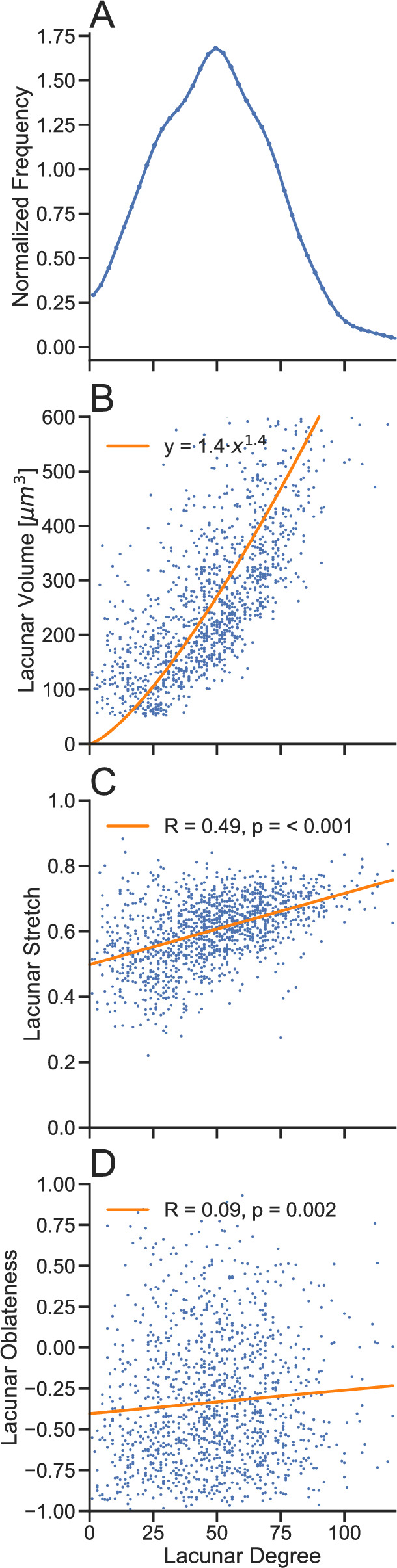
Analysis of the lacunae and their degree, i.e., number of canaliculi emanating from it, for the BALB/c mouse of Fig. 1 (average values with standard deviation over all mice (*n* = 5) are provided in the text). **A** shows the frequency distribution of the lacunar degree, **B**–**D** show correlations of lacunar volume and morphological parameters (stretch and oblateness) with lacunar degree. Voluminous lacunae and more stretched lacunae tend to have a higher degree, thus more canaliculi emanating from them. Values of lacunar stretch are roughly limited to a range between 0.3 and 0.8

We characterized the lacunocanalicluar network in right (non-loaded) and left (loaded) tibiae of five female 26-week-old BALB/c mice. Loading had no influence on the architectural properties of the LCN as shown in the detailed evaluation below. The evaluation of a specific example for non-loaded and loaded tibiae, respectively, is shown in the Figs. 1, 2, 3 and the corresponding Figs. S1–S3 in the Supplementary Material. The example of a loaded cortex is presented in the main text due to specific features highlighted by arrows and discussed in more detail later. The 3D architecture of the LCN was imaged in a whole cross-section within the tibial-midshaft (Figs. 1A and S1A). Based on these image stacks, maps of different network properties based on the analysis of subvolumes (see Materials and Methods) were obtained (Fig. 1B–E). Already the raw images (Fig. 1A) of the LCN show structural heterogeneities with a darker horse-shoe like band going around the cross-section. While the increased darkness points to a reduced canalicular density, the most distinguishing feature is the organization of the LCN: within the band, the canaliculi appear less ordered compared to surrounding areas, where the majority of the canaliculi is radially aligned forming a comb-like structure. Spatial maps of network parameters (Fig. 1B,C,D) show the heterogeneity of the LCN in BALB/c mice displaying again the horse-shoe like band running around the cortex with lower canalicular density (Can.Dn), lower average branching point (node) number (Nd.Nr), and lower average number of branches per branching point (node degree) (Nd.Dg) compared to areas outside of that band. This region of reduced network connectivity was observed in all evaluated BALB/c mouse samples with some differences of shape within the cortex ranging from horse-shoe like band to a full eccentric ring. These shape differences are influenced by the exact position of the cross-sectional cutting plane, e.g., in relation to the tibiofibular junction (see Supplemental Fig. 5). However, the overall area fraction of this unordered region does not correlate with the position of the cutting plane but remains rather stable. The reduced network connectivity is also reflected in the spatial analysis of lacunae (Fig. 1E), with lacunae of lower degree found within and along the band of low network density. The qBEI image (Fig. 1F) shows as well a spatial heterogeneity with again a band of lower Ca content spanning around the cortex. However, this band of low Ca content does not spatially coincide with the horse-shoe like structure of low network density, but is wider and more or less contains the latter.

### Canalicular Density is Mainly Driven by More Branching Points

The canalicular density of the network depends only on the number of branching points, the average number of branches emanating from a branching point (node degree), and the average tortuosity of the canaliculi since canaliculi between distant branching points are rare. Assuming the canalicular tortuosity is of minor importance, we asked the question whether the observed heterogeneities of the network are due to a higher spatial density of branching points and/or due to more branches emanating from the branching point. To answer this question, the spatial maps of LCN parameters of Fig. 1 were used to correlate different network parameters in the same spatial position (i.e., for the same subvolume). Figures 2A and S2A, respectively, shows the correlation of canalicular density and average node number for the tibia of Fig. 1 (one data point for each subvolume). The strong correlation with a correlation coefficient averaged over all five mice with non-loaded and loaded tibiae separated (*R*^non−loaded^ = 0.85 ± 0.02, *R*^loaded^ = 0.86 ± 0.01, no statistically significant difference *p* = 0.45) suggests that a higher network density is obtained by a higher number of branching points. However, also the correlation of canalicular density with average node degree (Fig. 2B) is clearly positive with an *R*^non−loaded^ = 0.73 ± 0.05 and *R*^loaded^ = 0.74 ± 0.02, respectively (no statistically significant difference *p* = 0.70). The conclusion that local increases in network density are mainly obtained by more branching points, but that these additional branching points are also the origin of more branches, is confirmed by the positive correlation between average node number and average node degree for all mice (Fig. 2C, *R*^non−loaded^ = 0.43 ± 0.02, *R*^loaded^ = 0.45 ± 0.04, no statistically significant difference *p* = 0.32).

### More Canaliculi Emanate from Larger and More Stretched Lacunae

Our network analysis allows to address the question of how the size and shape of the osteocyte lacunae correlate with the number of canaliculi emanating from the lacuna. Figures 3A and S3A, respectively, shows the lacunar degree distributions for the exemplary mouse of Fig. 1. The mean lacunar degree for this mouse is 47 and the corresponding distribution is broad (standard deviation of the distribution 25). This substantial intra-individual variability contrasts with the smaller inter-individual variability given by the average over all mean lacunar degree values for the non-loaded and loaded tibiae and the corresponding standard deviation, Lc.Dg^non−loaded^ = 50.8 ± 6.1, Lc.Dg^loaded^ = 41.4 ± 8.1, no statistically significant difference, *p* = 0.09). Evaluation of the lacunar volume yielded Lc.V^non−loaded^ = 356.1 ± 52.8 µm^3^, Lc.V^loaded^ = 327.9 ± 29.3 µm^3^, no statistically significant difference, *p* = 0.29. Correlation of the lacunar degree to the volume of the lacuna shows a clear positive correlation (Fig. 3B). The corresponding power law regression yields an exponent equal to 1.35, which is close to 1.5. This is the expected value assuming that the area per canaliculus on the surface of the lacuna is constant, since the volume is proportional to the third power of the “radius” of the lacuna and the surface proportional to the second power. Values for the lacunar stretch are more or less limited to the interval between 0.3 and 0.8 with average values Lc.St^non−loaded^ = 0.66 ± 0.01, Lc.St^loaded^ = 0.65 ± 0.03, no statistically significant difference, *p* = 0.71. The lacunar stretch correlates positively with lacunar degree (*R*^non−loaded^ = 0.45 ± 0.05, *R*^loaded^ = 0.42 ± 0.06, no statistically significant difference, *p* = 0.64), i.e., more elongated lacunae are the source of more canaliculi. The point cloud for the lacunar oblateness (Fig. 3D) is shifted toward negative values (Lc.Ob^non−loaded^ = − 0.38 ± 0.06, Lc.Ob^loaded^ = − 0.46 ± 0.09, *p* = 0.029) indicating rod-like and not plate-like lacunae with statistically significant lower values for lacunar oblateness in the loaded tibiae. No distinct correlation to lacunar degree was found (*R*^non−loaded^ = -0.02 ± 0.05, *R*^loaded^ = 0.06 ± 0.07, no statistically significant difference, *p* = 0.14).

### Inter-individual Variability of the LCN is Smaller than Intra-individual Variability

**Fig. 4:**
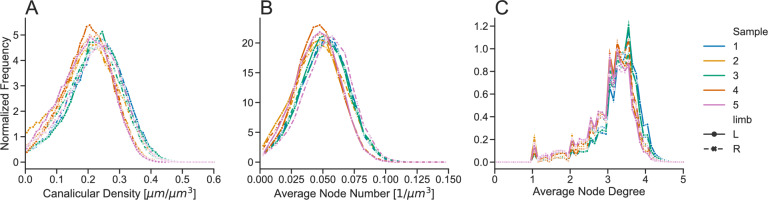
Frequency distributions for **A** canalicular density, **B** average node number and **C** average node degree for all examined BALB/c mice, right (R) tibiae non-loaded (dashed line with crosses), left (L) tibiae loaded (full line with full circles). For the frequency distribution all subvolumes in the imaged volume are considered. The distribution for Nd.Dg shows distinct peaks, which can be attributed to (i) the degree being by definition a natural number and (ii) some subvolumes containing only a small number of nodes. The maximum of nodes have a degree 3 ≤ Av.Nd.Dg ≤ 4. The inter-individual variability is much lower than the intra-individual variability for all parameters

The information content of the spatial maps of Can.Dn, Nd.Nr and Nd.Dg from Fig. 1 can be reduced into frequency distributions to assess the specific network parameters for all 10 tibiae of the five BALB/c mice (Fig. 4A–C). Similar to the results for lacunar degree, we observe that the inter-individual variability is much smaller than the intra-individual variability for those parameters: for each tibia a broad distribution is obtained, but these distributions are very similar for all 10 tibiae and are only slightly shifted with respect to each other. We quantify this by comparison of the standard deviation (SD) of the means with the mean of standard deviations of each distribution separating non-loaded and loaded limbs:

($\text{SD}\left({\text{Can}.\text{Dn}}_{\text{means}}^{\text{non}-\text{loaded}}\right)=0.009$ to $\text{mean}\left({\text{Can}.\text{Dn}}_{\text{SDs}}^{\text{non}-\text{loaded}}\right)=0.084$ and ($\text{SD}\left({\text{Can}.\text{Dn}}_{\text{means}}^{\text{loaded}}\right)=0.013$ to $\text{mean}\left({\text{Can}.\text{Dn}}_{\text{SDs}}^{\text{loaded}}\right)=0.082$, respectively. ($\text{SD}\left({\text{Nd}.\text{Nr}}_{\text{means}}^{\text{non}-\text{loaded}}\right)=0.003$ to $\text{mean}\left({\text{Nd}.\text{Nr}}_{\text{SDs}}^{\text{non}-\text{loaded}}\right)=0.019$ and ($\text{SD}\left({\text{Nd}.\text{Nr}}_{\text{means}}^{\text{loaded}}\right)=0.003$ to $\text{mean}\left({\text{Nd}.\text{Nr}}_{\text{SDs}}^{\text{loaded}}\right)=0.018$, respectively. ($\text{SD}\left({\text{Nd}.\text{Dg}}_{\text{means}}^{\text{non}-\text{loaded}}\right)=0.09$ to $\text{mean}\left({\text{Nd}.\text{Dg}}_{\text{SDs}}^{\text{non}-\text{loaded}}\right)=0.59$ and ($\text{SD}\left({\text{Nd}.\text{Dg}}_{\text{means}}^{\text{loaded}}\right)=0.14$ to $\text{mean}\left({\text{Nd}.\text{Dg}}_{\text{SDs}}^{\text{loaded}}\right)=0.60$, respectively.

### Loading Does Not Influence the LCN Architecture

**Fig. 5:**
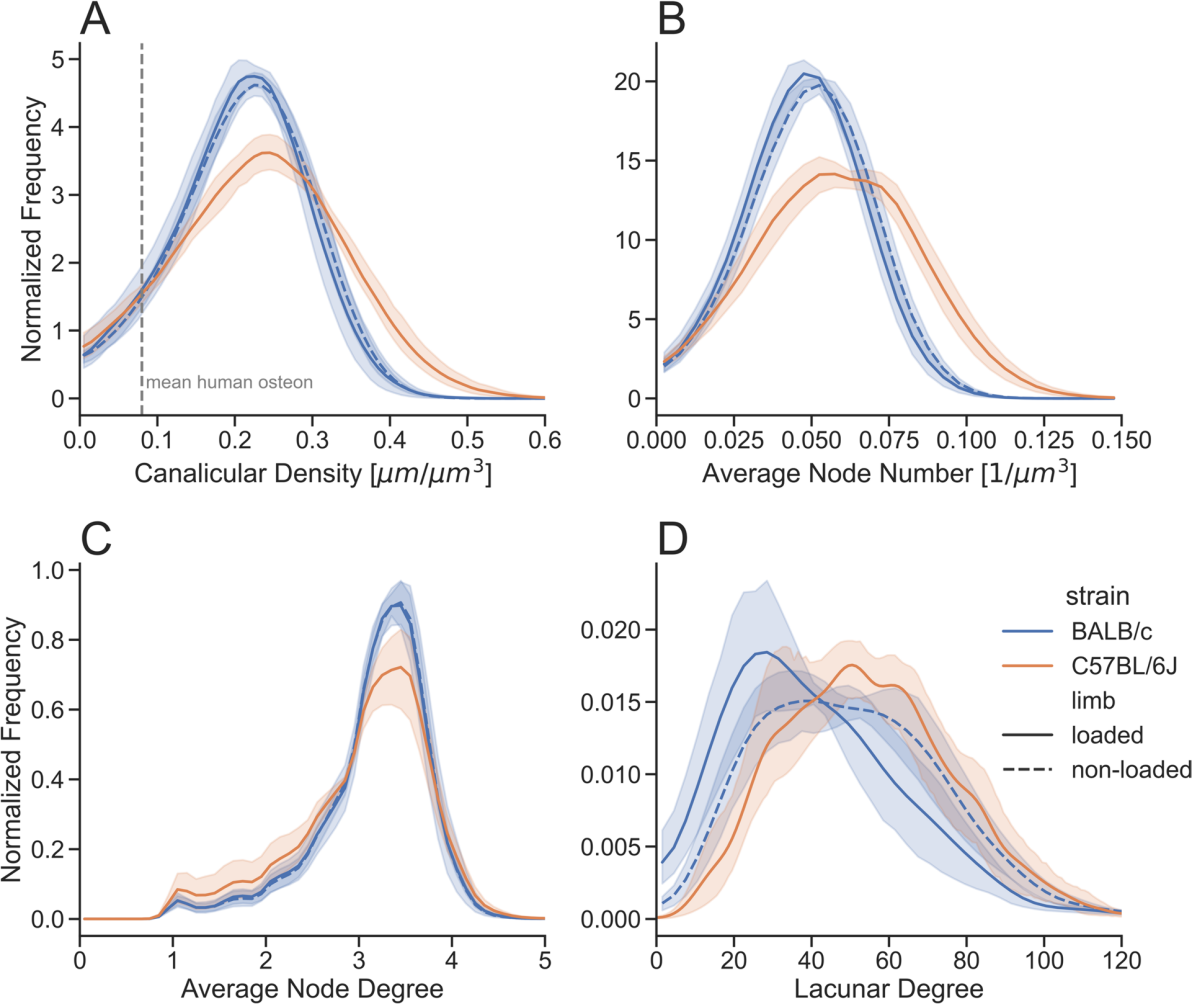
Network parameter distributions comparing the lacunocanalicular network between BALB/c (blue) and C57BL/6J (orange) mice, full line corresponding to the loaded tibia, dashed line to the non-loaded tibia, *n* = 5 in all groups. The histogram curves were smoothed by Gaussian filtering (*σ* = 1). The line shows the average values for the parameter and the shaded part displays the 95% confidence interval

We analyzed the LCN in the cortex of non-loaded and loaded limbs, where in the analysis only the pre-existing cortex was included; bone newly formed in response to the anabolic loading stimulus was excluded. Statistical tests failed to detect a significant difference in the LCN properties between the non-loaded and loaded limbs with the exception of lacunar oblateness. Furthermore, plotting the distributions for the network parameters canalicular density, average node number and average node degree for the non-loaded (dashed blue line) and the loaded limb (full blue line) results in almost indistinguishable curves (Fig. 5A–C). Evaluation of the corresponding mean values over the five animals does not show any significant differences: ${\text{Can}.\text{Dn}}_{\text{BALB}/\text{c}}^{\text{non}-\text{loaded}}=0.21\pm 0.009$ μm/μm^3^ and ${\text{Can}.\text{Dn}}_{\text{BALB}/\text{c}}^{\text{loaded}}=0.21\pm 0.013$ μm/μm^3^, *p* = 0.55; ${\text{Nd}.\text{Nr}}_{\text{BALB}/\text{c}}^{\text{non}-\text{loaded}}=0.050\pm 0.003$ μm^−3^ and ${\text{Nd}.\text{Nr}}_{\text{BALB}/\text{c}}^{\text{loaded}}=0.0.048\pm 0.003$ μm^−3^ *p* = 0.29; $\text{Nd}.\text{Dg}=3.16\pm 0.09$ and ${\text{Nd}.\text{Dg}}_{\text{BALB}/\text{c}}^{\text{loaded}}=3.15\pm 0.14$, *p* = 0.90.

### C57BL/6J Mice have a Denser Network Compared to BALB/c Mice

In the next evaluation step, we compare the LCN architecture in BALB/c and C57BL/6J (Fig. 5A–D). The mean value for canalicular density is higher in C57BL/6J mice compared to BALB/c mice, ${\text{Can}.\text{Dn}}_{\text{C}57\text{BL}/6\text{J}}^{\text{loaded}}=0.23\pm 0.01$ μm/μm^3^, *p* = 0.03 compared to loaded BALB/c and p = 0.02 compared to non-loaded BALB/c. The same significant difference can be observed for Nd.Nr (Fig. 5B), with C57BL/6J mice showing overall more branching points (nodes) per unit volume (${\text{Nd}.\text{Nr}}_{\text{C}57\text{BL}/6\text{J}}^{\text{loaded}}=0.059\pm 0.004$, μm^−3^, *p* = 0.002 compared to loaded BALB/c and *p* = 0.006 compared to non-loaded BALB/c). No significant differences were found between the two mouse strains in Nd.Dg (Fig. 5C) (${\text{Nd}.\text{Dg}}_{\text{C}57\text{BL}/6\text{J}}^{\text{loaded}}=3.05\pm 0.19$, *p* = 0.38 compared to loaded BALB/c and *p* = 0.29 compared to non-loaded BALB/c). The observation of a denser LCN in C57BL/6J mice is accurate also on the level of the single lacuna. The lacunar degree distributions in both strains differ significantly (Fig. 5D) when comparing the loaded tibiae, with C57BL/6J mice showing lacunae with more emanating canaliculi (${\text{Lc}.\text{Dg}}_{\text{C}57\text{BL}/6\text{J}}^{\text{loaded}}=55.4\pm 9.3$, *p* = 0.03, no statistical significance (*p* = 0.38) when comparing to non-loaded BALB/c). A closer look at the distributions of the network parameters (Fig. 5A–D) shows that for both Can.Dn and Nd.Nr, the distributions of C57BL/6J mice are shifted to higher values and are broader.

### Traces of Bone Development and Modeling can be Detected in the LCN

**Fig. 6:**
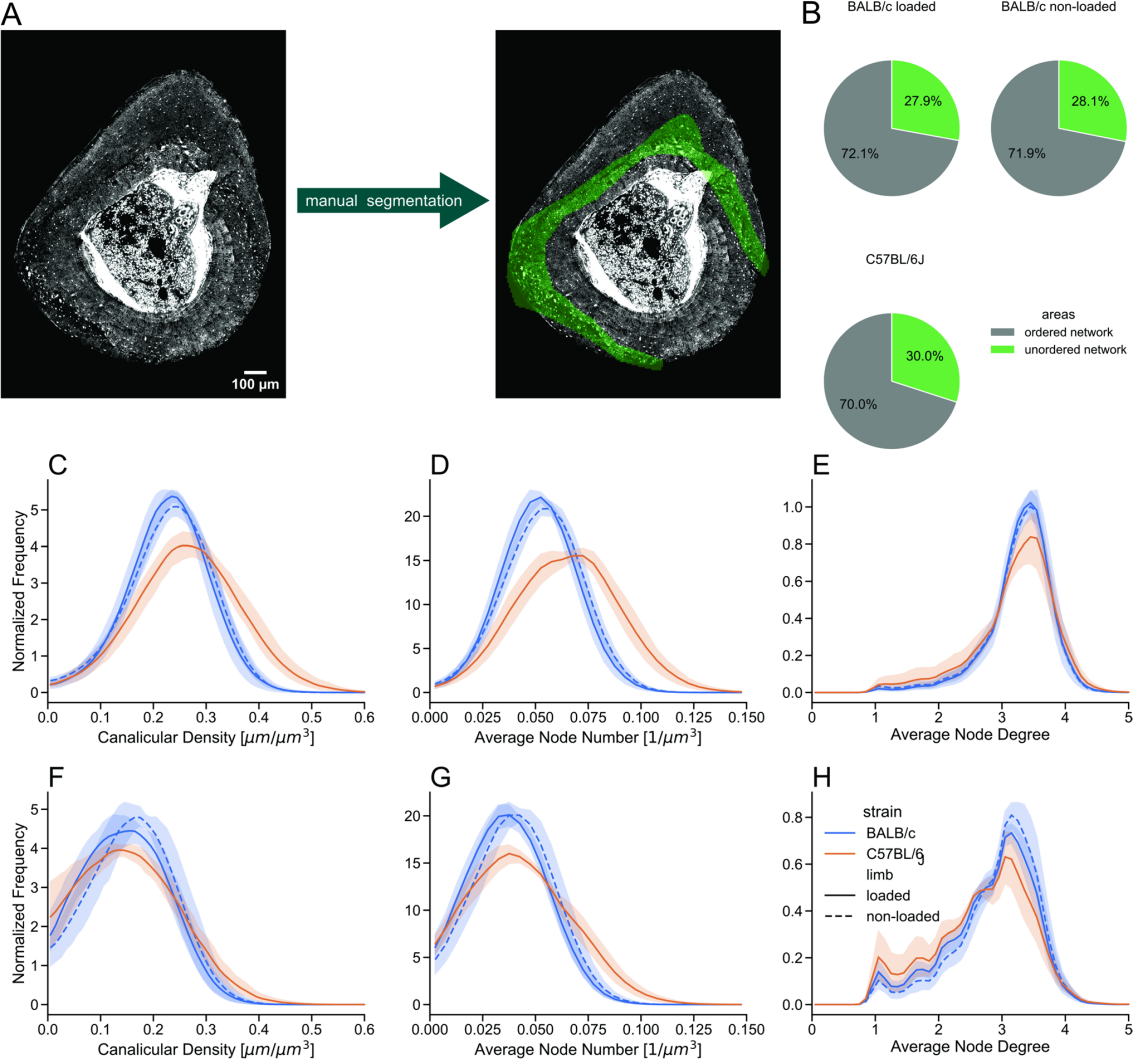
**A** The lacunocanalicular network in the murine cortices were separated based on the network organization into an ordered and unordered network (see the Materials and Methods section and Fig. S4 for more details). The manually found green mask highlights the region of unordered network. **B** mean area percentages of ordered and unordered network for both BALB/c and C57BL/6J mice. **C**–**E** comparison frequency distributions of network parameters in the ordered network region between BALB/c (blue) and C57BL/6J (orange) mice, full line corresponding to the loaded tibia, dashed line to the non-loaded tibia, *n *= 5 in all groups. The line shows the average values for the parameter and the shaded part displays the 95% confidence interval. **F**–**H** corresponding plots for the unordered network region

In both mouse strains a horse-shoe like band that sometimes closes to a full eccentric ring of unordered network with a lower density could be observed. Since this band corresponds to woven bone remaining from early bone development [46], we refined our analysis to ask the question whether differences in the LCN between the mouse strains can also be observed in this region of unordered network. We first ruled out that observed LCN difference between mouse strains are simply due to different volume fractions of unordered low-density network and ordered high density network. The volume fraction for the unordered network region is 28.1 ± 5.8% and 27.8 ± 3.0% in non-loaded and loaded BALB/c mice, respectively (*p* = 0.94) compared to 30.0 ± 6.5% in C57BL/6J loaded mice (*p* = 0.53 and *p* = 0.65 compared to loaded/non-loaded BALB/c mice). When looking at the normalized frequency distributions for unordered and ordered network separately, we observe first that the ordered network is denser than the disordered network, for both mouse strains and independent of the loading condition (${\text{Can}.\text{Dn}}_{\text{BALB}/\text{c }(\text{ord})}^{\text{non}-\text{loaded}}=0.23\pm 0.01$ μm/μm^3^ and ${\text{Can}.\text{Dn}}_{\text{BALB}/\text{c }(\text{unord})}^{\text{non}-\text{loaded}}=0.16\pm 0.02$ μm/μm^3^, *p* = 0.001), (${\text{Can}.\text{Dn}}_{\text{BALB}/\text{c }(\text{ord})}^{\text{loaded}}=0.23\pm 0.02$ μm/μm^3^ and ${\text{Can}.\text{Dn}}_{\text{BALB}/\text{c }(\text{unord})}^{\text{loaded}}=0.15\pm 0.02$ μm/μm^3^, *p* ≤ 0.001) (${\text{Can}.\text{Dn}}_{\text{C}57\text{BL}/6\text{J }(\text{ord})}^{\text{loaded}}=0.27\pm 0.02$ μm/μm^3^ and ${\text{Can}.\text{Dn}}_{\text{C}57\text{BL}/6\text{J }(\text{unord})}^{\text{loaded}}=0.16\pm 0.02$ μm/μm^3^, *p* ≤ 0.001). Similar to the evaluation of the whole network, the ordered network is significantly denser in C57BL/6J mice compared to BALB/c mice (loaded tibia: *p* = 0.02, Fig. 6C). The mean values of Can.Dn for the unordered network do not differ significantly (*p* = 0.40), but the corresponding distributions are broader for the C57BL/6J mice (Fig. 6F). The average node number shows similar behavior than the canalicular density (Fig. 6D and G), while the average node degree is not significantly different between the two strains (Fig. 6E and H).

Also, bone modeling leaves its trace in the structure of the LCN. A comparative observation between an image of the LCN and the map of the material composition (Ca content) allows conclusions about the temporal sequence of bone formation. The qBEI image shows a sharp interface between regions of very different Ca content (blue arrow in Fig. 1F). This interface also shows up in the LCN image as almost a complete disruption of the network. While the murine cortex is not remodeled as the human cortex, changes in the intracortical structure are not excluded. The backscattered electron image shows traces of a blood vessel canal drifting through the cortex. This drift appears in the LCN as an intriguing fan-like architecture, which is well separated from the rest of the canalicular network (Fig. 1A and F, yellow arrow).

### The Node Degree Distribution Decays Exponentially

**Fig. 7:**
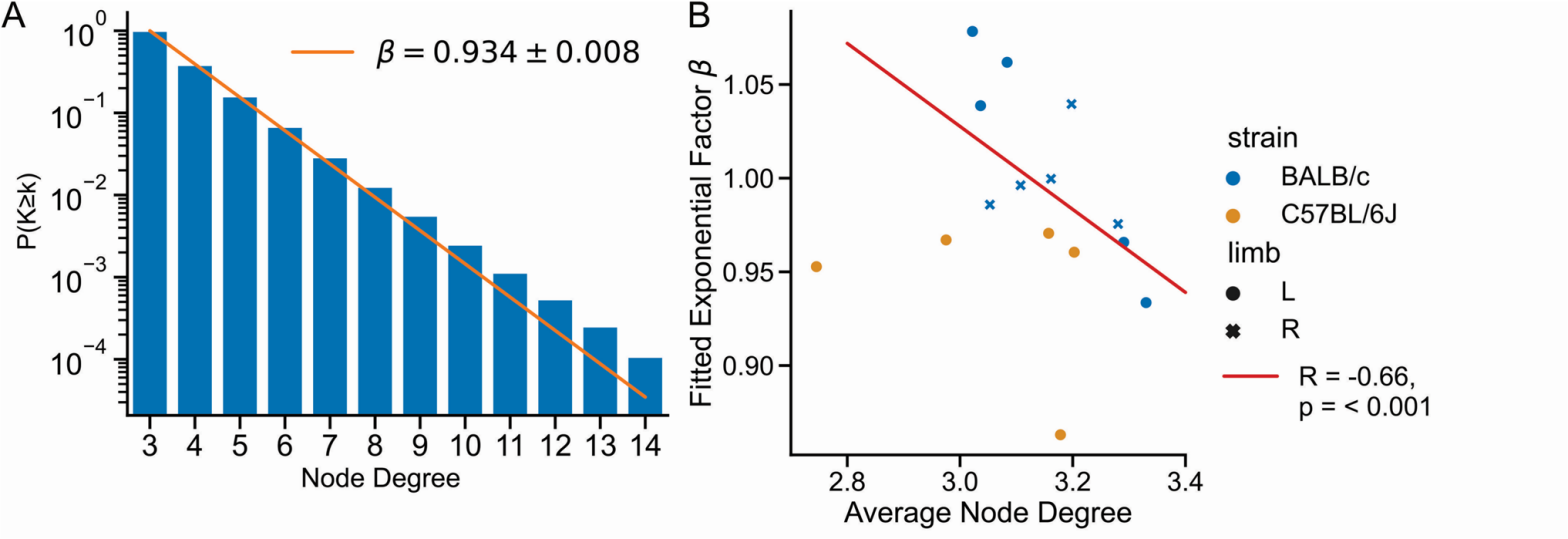
**A** Semi-logarithmic plot of the cumulative node degree distribution for one BALB/c mouse, which provides information about the percentage of nodes with a certain degree or larger. The node degree distribution was found to decay approximately exponentially with an exponent β of around 1. Most nodes have degree 3. **B** The mean average node degree of all BALB/c mice plotted against the respective fitted exponential factor as well as a linear regression fit (red line). With increasing mean average node degree the node degree distribution decays more slowly

The degree of nodes in the network can be assessed without partitioning of the imaged volume into subvolumes. We evaluated the cumulative node degree distribution, i.e., the proportion of nodes that have a degree larger than a certain value, in both BALB/c and C57BL/6J mice, performed a fit of the decay with an exponential function ${e}^{-\beta (x-3)}$ following [42] and scrutinized the obtained fitting exponents (Fig. 7). The values of the exponent were found to be very close to 1 for both mouse strains. A correlation between each individual fitting exponent β with the corresponding mean average node degree yielded a negative correlation (Fig. 7B). Data points for the C57BL/6J are found closer to the origin of the plot with small values for both the exponent β and mean average node degree.

## Discussion

In this study we report an extensive quantification of the spatial heterogeneity of the lacunocanalicular network (LCN) in adult 26-week-old BALB/c and compare obtained results to the LCN from C57BL/6J mice of the same age. We correlate spatial maps of different network parameters to identify the essential contributors to the spatial intra-individual variability of network density. The most important contributor for a locally denser network turned out to be the local increase in the number of branching points within the network. Of less importance, with a lower correlation coefficient, is the branching, the number of canaliculi radiating from the branching point. However, the positive correlation between the number of branching points and number of branches (Fig. 2C) shows that both network parameters increase simultaneously to obtain a denser network.

Loading had no influence on the architectural properties of the LCN when the analysis was restricted to the pre-existing cortex excluding the bone newly formed as a response to loading. A contrary result would have been unexpected since it would have indicated that the LCN is able to adapt its architecture on a time scale of only two weeks. In the comparison between BALB/c and C57BL/6J mice it would have been preferable also to include the non-loaded limbs of the C57BL/6J mice. Sample damage, most likely due to some unwanted dehydration, resulted in a reduced image quality, which did not allow to analyze the LCN in large parts of the cortex of three mice. For the two remaining samples, which could be evaluated, we checked that obtained results are indistinguishable from the loaded group, but we did not include any of this data in our evaluation for reasons of statistics.

In both studied mouse strains, BALB/c and C57BL/6J mice, the LCN in the tibia can be decomposed into an unordered network of lower density and an ordered network of higher density. The unordered network is arranged in an intracortical horse-shoe like band, which coincides with woven bone including cartilage islands [46, 47]. A comparison between the two mouse strains showed that C57BL/6J mice have an overall denser network. This difference cannot be explained by different area fractions of ordered and unordered bone. In both mouse strains about 30% of the bone volume in the tibial-midshaft the LCN is unordered. Comparing the two mouse strains, C57BL/6J showed regions of higher canalicular density and higher density of branching points in the region of unordered network. Since the region of the unordered network corresponds to the early development of murine cortical bone, the LCN is different between the two mouse strains from a very early time point of bone development. Moreover, the LCN was found to be denser in C57BL/6J mice in the ordered network region corresponding to more mature bone. The overall higher network density of the LCN in C57BL/6J mice is also reflected locally in the network around a lacuna. On average, more canaliculi are emanating from a lacuna in C57BL/6J mice compared to BALB/c mice.

Our values found for the lacunar degree are slightly larger than the ones reported in a study which used the C57BL/6 mouse model to investigate the effect of aging. About 40 canaliculi emanated on average from a lacuna for female mice (5 months) and this value dropped dramatically by 45% in female aged mice (23 months) [48]. In cortical bone of humans (female donors, 56–95 years old, femoral diaphysis) an average value of 58.2 primary canaliculi per lacuna was found [13]. The largest lacunar degree was reported in rats, with 83.9 in female Sprague Dawley rats (26 weeks) [49] and near to 80 in male Wistar rats (17 weeks) [50].

Our investigations reveal a very high intra-individual variability of LCN properties, which contrasts with a rather low inter-individual variability. This is best demonstrated in Fig. 5 with broad frequency distributions, e.g., for canalicular density, but all these distributions are similar for all ten investigated tibiae of BALB/c mice. The intra-individual variability is not simply a consequence of the composition of the network in an ordered and unordered part. While the network density is lower in the unordered network compared to the ordered network, each component of the LCN taken by itself is highly variable in density (Fig. 6). This variability extends to the local network level with a strongly variable number of canaliculi emanating from a lacuna (Fig. 5D). The high intra-individual variability of the LCN implies that caution must be taken when selecting regions of interest (ROI) for LCN characterization. ROIs that cover only small parts of the cortex run the serious risk that they do not include a representative volume of both ordered and unordered network. In the case that the investigated volumes include only a few lacunae, results, for example, for lacunar degree can easily differ by more than 100%, although selected volumes are only 50 µm apart. Our recommendation is to image the LCN in transversal sections and to try to characterize the LCN in (almost) the whole cross-section to ascertain a more representative measure of the cortical bone. Future research is required to determine if a smaller region of the LCN can serve as a representative proxy for the overall network properties.

Two important questions to be asked concern dynamic information on bone and network formation: what can we learn about bone modeling from the architecture of the LCN; and how much information can be obtained from a quantitative network analysis about the network formation itself. In addressing the first question, the qBEI image (Fig. 1F) helps since it outlines the long bone development as initial formation of a ring (in the two-dimensional cross-section) of smaller size and lower calcium content (darker colors in Fig. 1F). On this ring bone apposition can occur both on the endocortical and periosteal surface. This later deposited bone is higher mineralized resulting in a rather sharp interface running through the cortex (marked by a blue arrow in Fig. 1F). These two separate phases found in the cortex are corroborated by the corresponding images of the LCN. At the interface between lower and higher mineralized bone, the network displays a severe discontinuity with hardly any canaliculi crossing the interface. This view that (re)modeling events leave a trace of disruption in the LCN is confirmed by the network around the vascular canal in the upper-left part of the cortex close to the endocortical surface. The qBEI image suggests a drift [51] of the vascular canal toward the endocortical surface. In the LCN image this drift of the canal leaves behind a curiously organized fan-shaped network, which is again markedly isolated from the surrounding LCN. Intracortically, no network disruptions are expected since mice only in rare circumstances exhibit intracortical remodeling [30, 52]. However, in humans it is known that only a few canaliculi cross the cement lines of osteons [53, 54]. Thus, it might be possible to reconstruct the succession of bone remodeling events by analyzing discontinuities in the canalicular network structure.

The second question, how much can be learned from the network architecture regarding the formation process of the network itself is difficult to address. In this context the most important observation of our study seems that the branching of the network does not differ between BALB/c mice and C57BL/6J mice. Most of the nodes of the network are intersection points between canaliculi, and in most of the cases (70%) three canaliculi come together in a branching point. The LCN in mice is a tree-like network, i.e., it is dominated by network nodes of degree three [42].

Can the LCN architecture be modified after its initial formation? We speculate that cell processes of pre-osteocytes, which are already partly embedded in mineralized matrix and are in contact with the cell processes of bone forming osteoblasts at the surface, have the ability to modify the LCN by forking into two. Another example of post-formation modifications of the LCN architecture could be the so-called canalicular junctions reported by Wittig et al. [55]. These cavities are less than 30 µm^3^ in volume (i.e., much smaller than an osteocyte lacuna) and have on average 7 canaliculi emanating from them. Canalicular junctions could be formed in mature bone in a later process, where closely neighboring canaliculi dissolve the matrix which is separating them. Such a matrix resorption via osteocytic osteolysis has been reported multiple times [9, 56–58] and is corroborated by osteocytes being TRAP positive [59, 60]. One reason why discussions about network formation and modifications remain highly speculative is that our methodology images the lacunocanalicular network only, but does not give information on the cell network of osteocytes, the driving force behind modifications. The research group of Sarah Dallas [48, 61] has used methods that stain both networks, the osteocyte network and the LCN. For C57BL/6 mice they reported that only about 80% of the canaliculi are occupied by cell processes, a number which decreases with aging.

In our study the left tibiae of the mice underwent a controlled in vivo loading. The loading protocol elicited an anabolic response in adult (26-week-old) female C57BL/6J mice [33], but not in age and sex matched BALB/c mice at a strain level of 1200 µε (determined via strain gauging of the mid-diaphysis). Rather, a strain level of 1800 µε elicited an anabolic response in 26-week-old female BALB/c mice [32]. Since our study aimed to determine if short term anabolic loading resulted in altered LCN architecture of the pre-existing mature cortical bone, we used different strain magnitudes on the different genetic mouse strains. The loading of the left tibia had no detectable effect on the quantified network parameters. An important next step is to relate network architecture with the fluid flow through the network to conclude about the local mechanosensitivity of the bone. If the fluid flow through the LCN would be provoked exclusively by an externally applied pressure difference, then a denser network should have lower flow resistance. As a consequence a denser network would result in a faster fluid flow and thus more shear and drag forces on the osteocyte dendritic processes, which in turn stimulates osteocytes [3, 6, 19, 62]. However, the fluid flow through the LCN is load-induced, i.e., the cause for the fluid to flow is a volume change of the LCN due to the loading. Studies on human osteons based on load-induced fluid flow demonstrated rather counter-intuitively that a LCN with compromised connectivity can nevertheless produce a very effective fluid flow [53]. Consequently, only a detailed fluid flow analysis can provide a definitive answer of how the changes in network architecture between BALB/c mice and C57BL/6J mice translate into different mechanoresponses. In terms of transport properties, a denser network should make the delivery of nutrients and the removal of waste products more efficient. Also mineral precursors have to be transported toward the mineralization site by the LCN [17, 63]. Here one can speculate that the denser network in C57BL/6J mice results in a more efficient transport of mineral precursors and consequently to a faster mineralization kinetics compared to BALB/c mice. In conclusion, we assessed the spatial heterogeneity in the LCN of BALB/c mice and compared outcomes to those obtained in C57BL/6J mice. We found a denser network in C57BL/6J and could show that this can be attributed to an increased number in branching points of the network. Our findings will inform future studies about the importance of assessing wider regions of the LCN and will help the understanding of the difference in mechanoresponse in different mouse strains.

## Supplementary Information

Below is the link to the electronic supplementary material.

Supplementary file1 (PDF 3505 KB)
