# Supplementary material for: The Lacunocanalicular Network is Denser in C57BL/6 Compared to BALB/c Mice
Source: Calcif Tissue Int. 2024 Oct 16;115(5):744–58. doi: 10.1007/s00223-024-01289-y (PMC11531440; doi:10.1007/s00223-024-01289-y)
Supplement: Supplementary file 1 — Supplementary file1 (PDF 3505 KB) [file 223_2024_1289_MOESM1_ESM.pdf]

The lacunocanalicular network is denser in C57BL/6 compared to BALB/c mice

Maximilian Rummler[1], Alexander van Tol[1], Victoria Schemenz[1], Markus Hartmann[2], Stephane Blouin[2], Bettina M. Willie[3] and Richard Weinkamer[1]

Supplementary Material

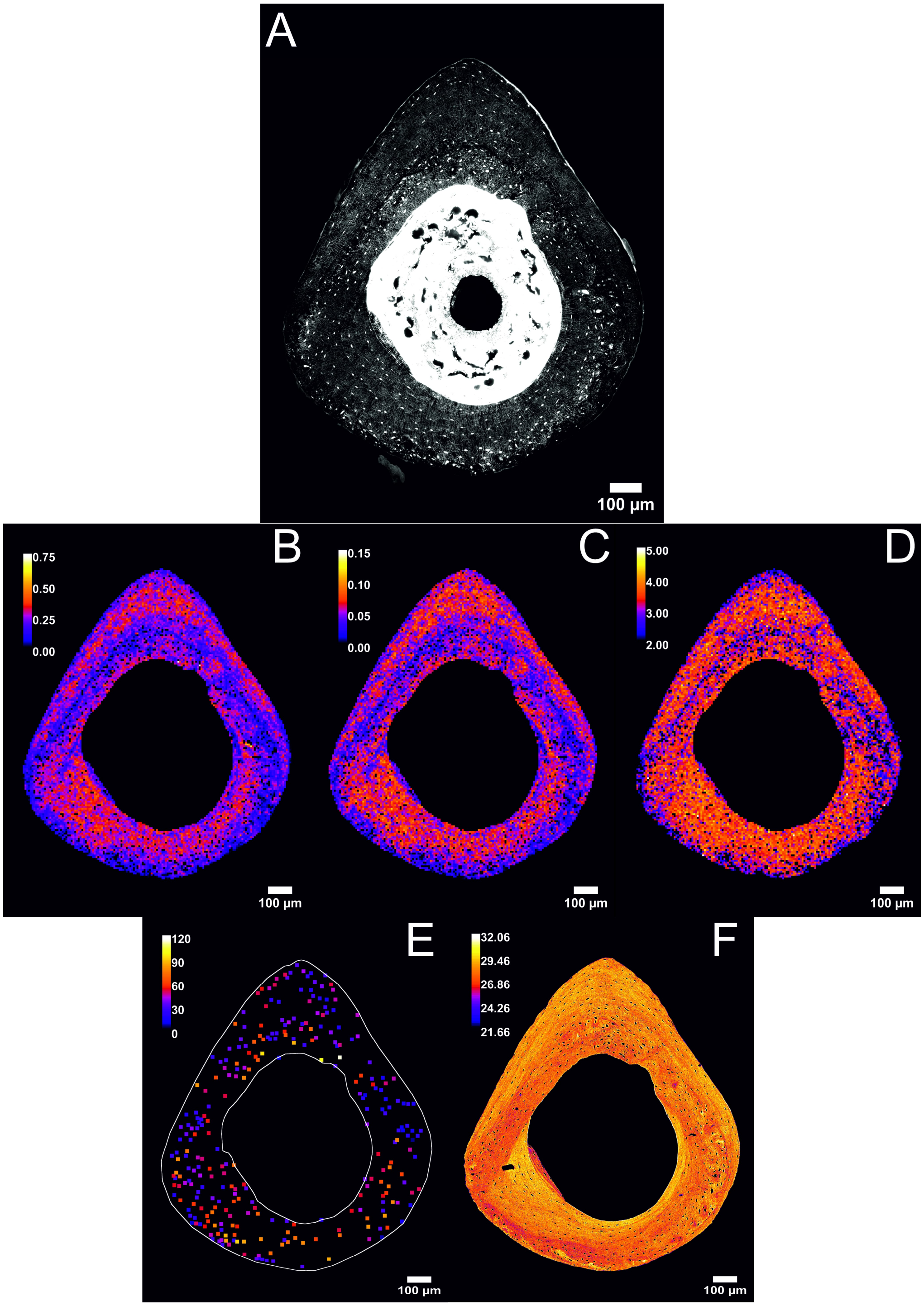

Supplemental Figure S1: Lacunocanalicular network within a tibial cross section of a BALB/c mouse, right, non-loaded tibia. A) shows a single image of an image stack obtained by confocal microscopy where bright dots correspond to osteocyte lacunae. B) – E) show maps of network parameters obtained by evaluation of network properties within 400 µm^3 cubic subvolumes: B) canalicular density as length of the canaliculi per volume [µm/µm3], C) the average node number, i.e. the number of branching points of the network per volume [#/µm3], D) the average node degree, i.e., the average number of branches emanating form a branching point; E) an intensity projection along the z-axis of 3.4 µm of lacunae and their degree, i.e. the number of canaliculi emanating from the lacunae. The white line shows the outline of the tibial cross-section. F) the Ca content [wt%Ca] measured by quantitative Backscattered Electron Imaging (qBEI).

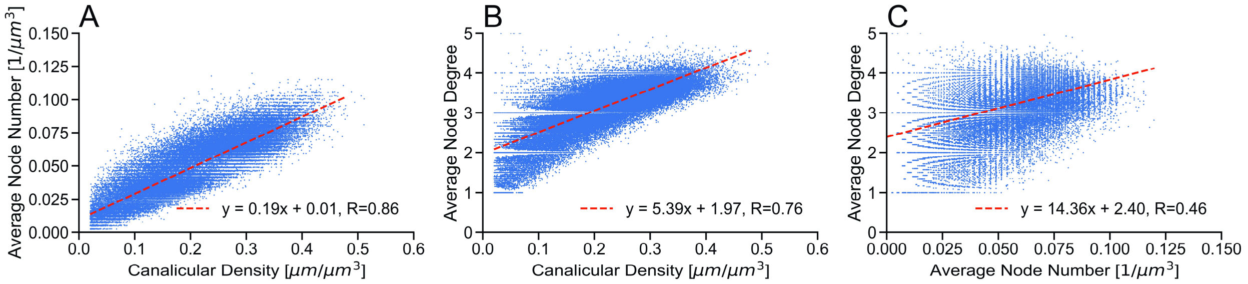

Supplemental Figure S2: Correlation plots of network parameters for the BALB/c mouse of Fig. S1, right, non-loaded, tibia. A) shows the correlation between canalicular density and average number of branching points (average node number), B) the correlation between canalicular density and average number of branches emanating from a branching point (average node degree) and C) the correlation of average node number and average node degree. The red line denotes a linear least squares regression; given is slope and intercept of the straight line, together with the Pearson correlation coefficient R. Patterns in the arrangement of data points as seen in Fig. 2C are due to subvolumes with only a few nodes and the mode of plotting, which spreads identical data points.

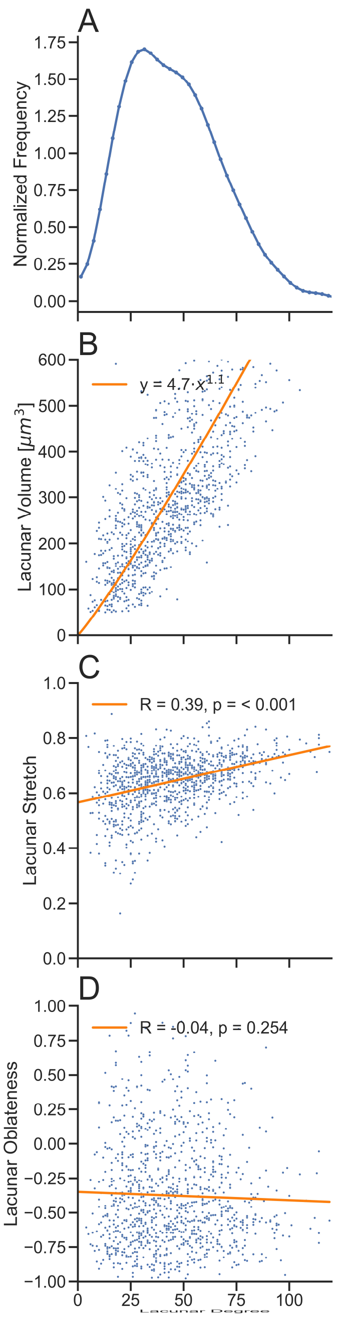

Supplemental Figure S3: Analysis of the lacunae and their degree, i.e., number of canaliculi emanating from it, for the BALB/c mouse of Fig. S1. A) shows the frequency distribution of the lacunar degree, B-D show correlations of lacunar volume and morphological parameters (stretch and oblateness) with lacunar degree. Voluminous lacunae and more stretched lacunae tend to have a higher degree, thus more canaliculi emanating from them. Values of lacunar stretch are roughly limited to a range between 0.3 and 0.8.

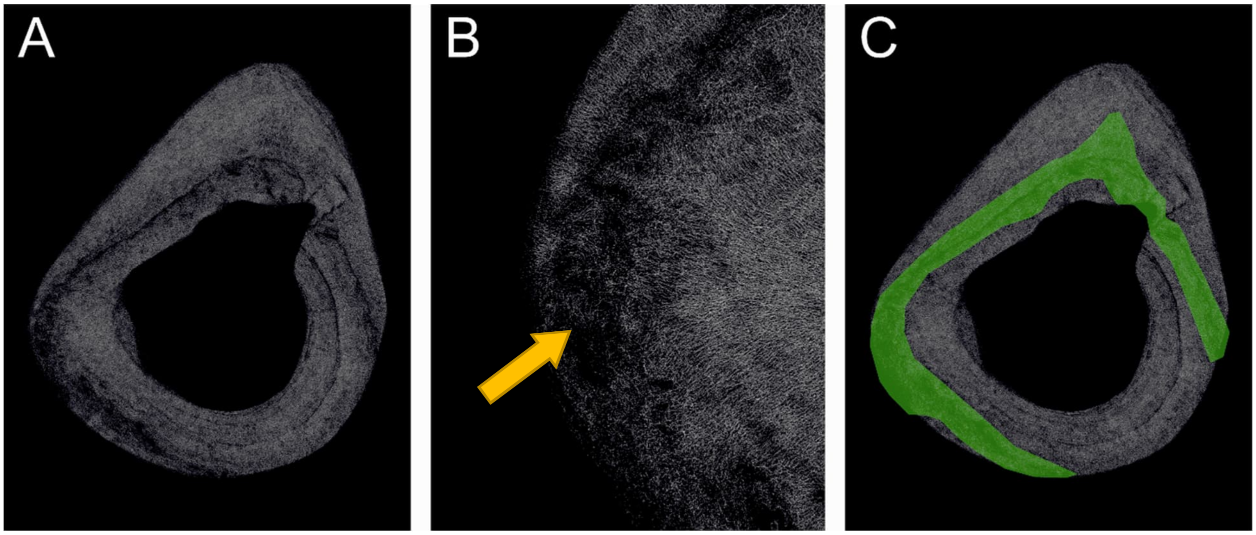

Supplemental Figure S4: Shown is the identification of the unordered network region based on A) a projected image along the z-axis of the skeletonized dataset. B) shows the whirl-like structure of the unordered network (yellow arrow), compared to the comb-like structure of the ordered network. C) shows the overlap of the unordered network mask with the projection of the skeletonized network.

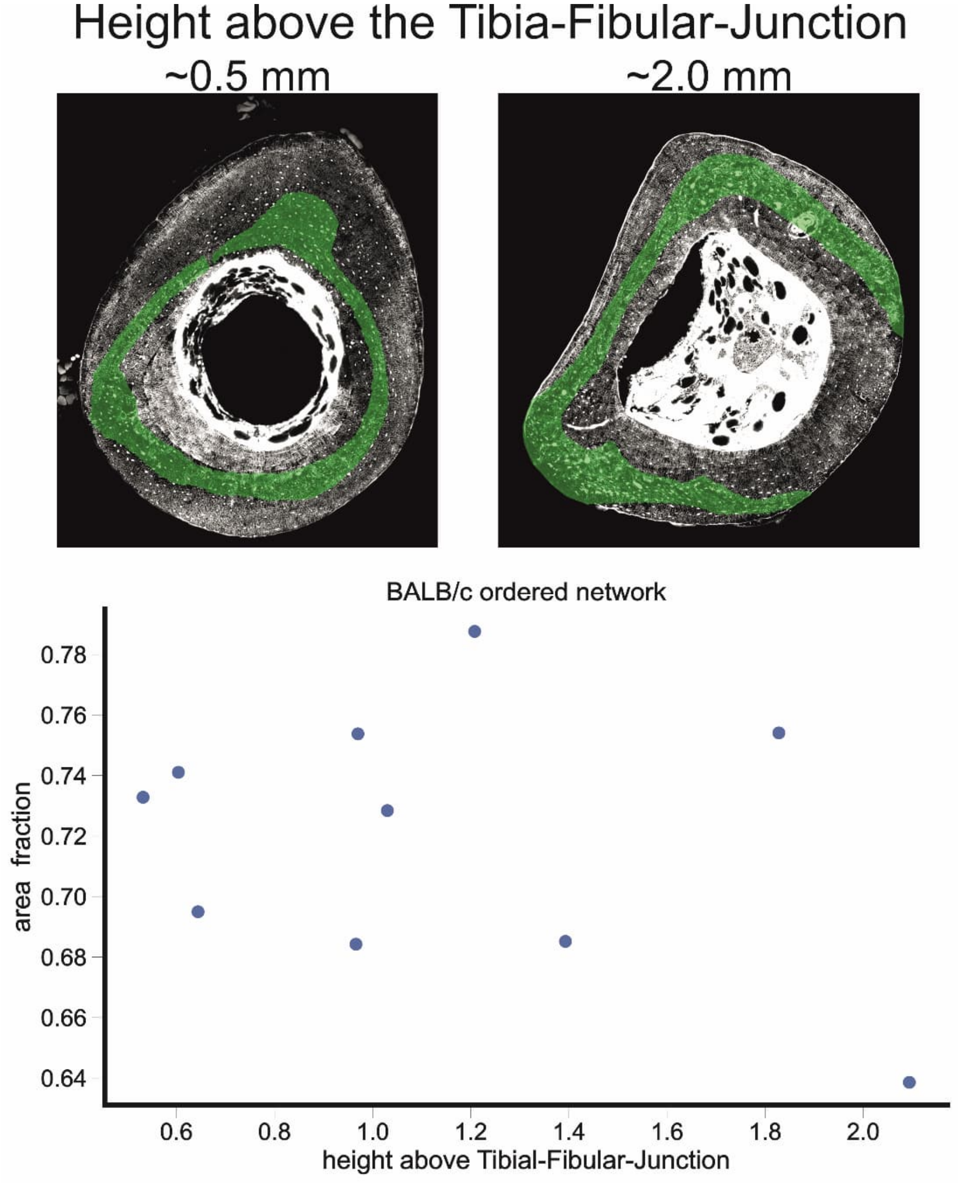

Supplemental Figure 5: Shown is the dependence of the shape of the unordered network region on the height above the tibia-fibular-junction (TFJ). Around 0.5 mm above the TFJ, the unordered region forms a ring around the cortex, while it changes with increasing height to a horseshoe like shape at 2 mm above the TFJ. The area fraction of the ordered network region does not show a correlation with the height of the cutting plane above the TFJ.

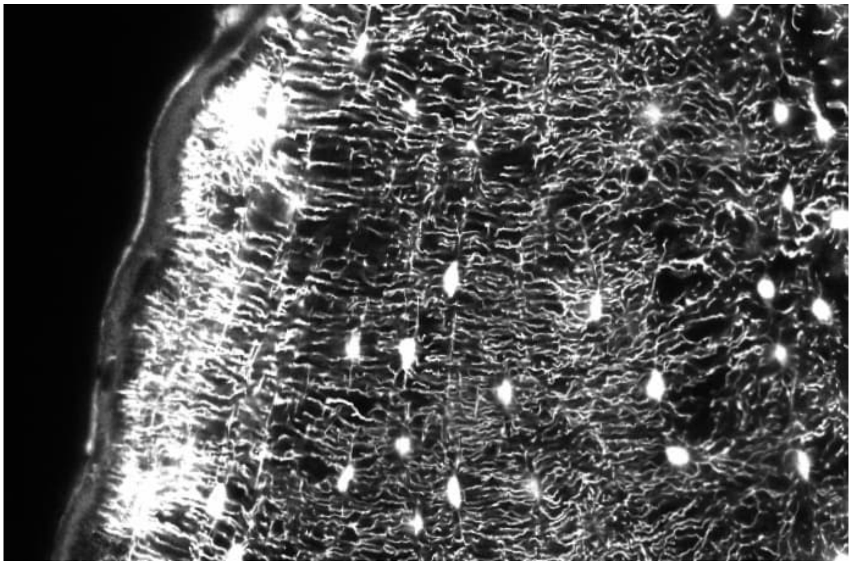

Supplemental Figure 6: Image of the lacunocanalicular network in a BALB/c mouse obtained with confocal microscopy after rhodamine staining. The detail shows part of the mouse cortex of a loaded tibia (periosteal surface to the left). As a response to the applied in vivo loading, new bone was formed at the periosteal surface. This newly formed bone is lowly mineralized allowing rhodamine to enter deeper into the bone matrix. The result is an overstaining of the network in large parts of the newly formed bone. Consequently, the newly formed bone was excluded from all evaluations.
